# Exploring the diversity of Poaceae-infecting mastreviruses on Reunion Island using a viral metagenomics-based approach

**DOI:** 10.1038/s41598-019-49134-9

**Published:** 2019-09-03

**Authors:** Sohini Claverie, Alassane Ouattara, Murielle Hoareau, Denis Filloux, Arvind Varsani, Philippe Roumagnac, Darren P. Martin, Jean-Michel Lett, Pierre Lefeuvre

**Affiliations:** 1 CIRAD, UMR PVBMT, F-97410, St Pierre, La Réunion, France; 2 Université de La Réunion, UMR PVBMT, Pôle de Protection des Plantes, 7 Chemin de l’IRAT, Saint-Pierre, 97410 France; 3 INERA, 01 BP 476, Ouagadougou 01, Burkina Faso; 4 Laboratoire Biosciences, Université Joseph KI-ZERBO, 03 BP 7021, Ouagadougou 03, Burkina Faso; 5 The Biodesign Center for Fundamental and Applied Microbiomics, Center for Evolution and Medicine, School of Life Sciences, Arizona State University, 1001 S. McAllister Ave, Tempe, AZ 85287-5001 USA; 6 Structural Biology Research Unit, Departement of Integrative Biomedical Sciences, University of Cape Town, Observatory, Cape Town, South Africa; 7 CIRAD, UMR BGPI, F-34398, Montpellier, France; 8 BGPI, Université de Montpellier, INRA, CIRAD, Montpellier SupAgro, F-34398, Montpellier, France; 9 Computational Biology Division, Departement of Integrative Biomedical Sciences, Institute of Infectious Diseases and Molecular Medicine, University of Cape Town, Observatory, South Africa

**Keywords:** High-throughput screening, High-throughput screening, Metagenomics

## Abstract

Mostly found in Africa and its surrounding islands, African streak viruses (AfSV) represent the largest group of known mastreviruses. Of the thirteen AfSV species that are known to infect either cultivated or wild Poaceae plant species, six have been identified on Reunion Island. To better characterize AfSV diversity on this island, we undertook a survey of a small agroecosystem using a new metagenomics-based approach involving rolling circle amplification with random PCR amplification tagging (RCA-RA-PCR), high-throughput sequencing (Illumina HiSeq) and the mastrevirus reads classification using phylogenetic placement. Mastreviruses that likely belong to three new species were discovered and full genome sequences of these were determined by Sanger sequencing. The geminivirus-focused metagenomics approach we applied in this study was useful in both the detection of known and novel mastreviruses. The results confirm that Reunion Island is indeed a hotspot of AfSV diversity and that many of the mastrevirus species have likely been introduced multiple times. Applying a similar approach in other natural and agricultural environments should yield sufficient detail on the composition and diversity of geminivirus communities to precipitate major advances in our understanding of the ecology and the evolutionary history of this important group of viruses.

## Introduction

Members of the *Mastrevirus* genus, one of the nine genera in the *Geminiviridae* family^1^, cause diseases of economically important crops such as maize^2^, wheat^3^, sugarcane^4^ and chickpea^5^. These viruses have ~2.5–2.7 kb circular single-stranded DNA genomes and are transmitted by leafhoppers (in the Cicadellidae family) to a range of either monocotyledonous or dicotyledonous host species.

Most of the known monocot-infecting mastreviruses have been identified either in Africa or surrounding islands. These mastreviruses have collectively been called the “African streak viruses” (AfSV). There are presently thirteen recognised AfSV species, nine of which have been found infecting both cultivated and non-cultivated host species, four of which have only been found infecting non-cultivated grasses^6^.

Viruses that infect cultivated plants represent only a small fraction of ecosystem level viral diversity^7^. As with many other groups of viruses, only a small fraction of the mastreviruses that currently exist has been discovered^7–9^. It is very likely that large numbers of presently unknown viruses reside within the thousands of non-cultivated and largely unsampled plant species that are found within the unmanaged portions of terrestrial environments. The significance of non-cultivated plant species both in the functioning and maintenance of virus communities, and in the emergence of new viral pathogens, has remained largely unexplored. This is in part due to the fact that many non-cultivated species display no easily identifiable symptoms when they are virus-infected^10,11^, and partly because of the prohibitive cost and effort that is required to acquire sufficient viral genomic sequence data from large-enough numbers of plants to obtain a global view of viral diversity at the ecosystem scale.

The development and widespread use of rolling circle amplification (RCA) based protocols for the recovery of complete mastrevirus genome sequences has vastly increased the feasibility of identifying viruses in large numbers of non-cultivated plant samples^6^. RCA has facilitated both the discovery of many new mastreviruses, and the characterization of diverse strain groups within already known species. For example, although the “A-strain” of maize streak virus (MSV-A), which causes severe disease in maize, was characterized in the early 1980s and four other “grass-infecting” strains were characterized during the 1990s following extensive sampling of non-maize grass species, RCA-based appoaches enabled the discovery of six additional MSV strains in a single small study^12^. Moreover, characterization of these additional strains revealed that the last common ancestor of all known MSV-A isolates was likely a recombinant of two “grass adapted” MSV-variants: one belonging to the MSV-B strain and the other most closely related to the MSV-F and MSV-G strains^12^. Although there is no direct proof that this recombination event triggered the emergence of MSV-A as a maize pathogen, its discovery highlights once again the importance of considering virus diversity at the ecosystem scale rather than at the scale of a single pathosystem. Similar discoveries for viruses in other families are shifting the global plant disease paradigm and have highlighted the utility of studying the whole viral communities (virome) within the context of their biotic environments (pathobiomes) to achieve a better understanding of how, for example, crop pathogens emerge from largely hypovirulent natural viral communities^13,14^.

Concomitant to, and in some cases driving our present perception of the roles that plant-viruses play in global ecosystems, has been the development and refinement of plant virus metagenomics techniques which enable the characterization of entire viral communities at ecosystem scales^9,15^. A growing number of methodologies have been developed that are based on the high throughput sequencing of total nucleic acids, nucleic acids from purified virus-like particles, or enriched virus-specific nucleic acids. By strategically tagging samples to preserve information on their host origins, one approach, called “eco-genomics”^11^, has proven extremely fruitful in the discovery of hundreds of new plant-virus associations within natural ecosystems^16^. However, the main challenge facing the widespread application of such metagenomics protocols remains their cost and the technical challenges inherent of acquiring and analysing the massive data sets that are generated.

Here, by focusing solely on viruses with circular DNA genomes, the power of RCA has been harnessed to enrich for circular viral nucleic acids and, in so doing, avoid many of the cumbersome aspects of ecogenomics approaches. The metagenomics approach used here is easy to implement and involves denser pooling of samples than in previous studies, while still yielding enough viral genomic sequence data to enable the study of virus diversity at both the individual plant and ecosystem scales. In addition, along with a conventional similarity search procedure for read assignment, a statistical phylogenetic placement methodology was used to classify virus sequence reads. As a proof of concept, this new metagenomic methodology was applied to a set of non-cultivated plants sampled from a single farm on Reunion Island. In addition to finding viruses that belong to two of the six mastreviruses species that have previously been detected on Reunion Island, MSV and maize streak Reunion virus (MSRV)^6,17^, this article further highlights the gaps that likely remain in our appreciation of mastrevirus diversity on this island by discovering mastreviruses that could represent three new mastrevirus species.

## Methods

### Sampling

Leaf samples of Poaceae plants (n = 144) were randomly collected regardless of whether they were symptomatic or asymptomatic on a 1000 m^2^ fallow plot at the Bassin Plat CIRAD experimental facility (Latitude −21.3231; Longitude 55.4912) in Saint Pierre (Reunion Island) during November 2014 (see Supplementary Table S1 for details). Only two of the collected plants presented visible typical symptoms of streak disease. Samples were dried in an oven at 50 °C overnight and stored at room temperature before use.

### Metagenomic approach

**Figure 1:**
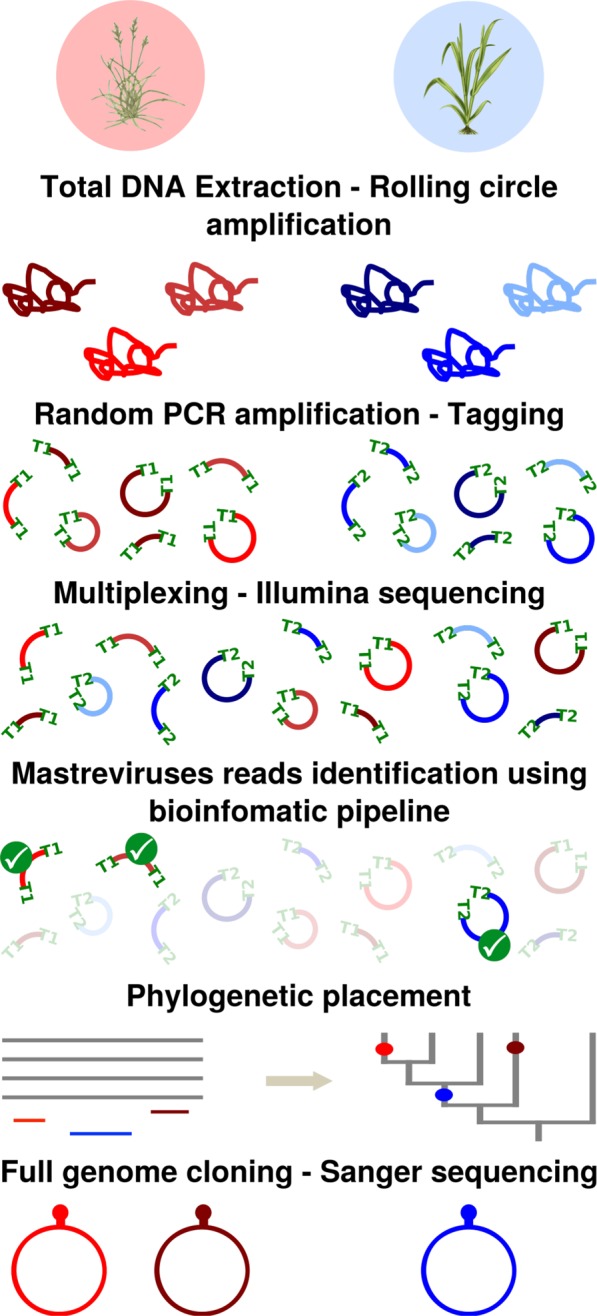
Schematic representation of the metagenomic approach. Total genomic DNA was extracted from dried leaf material, followed by a rolling circle amplification (RCA). A random amplification step using polymerase chain reaction (RA-PCR) combined with a tagging was performed. The use of distinct tags, here symbolized with the «T1» and «T2» labels, allows each sequence to be traced back to the original sample. During the multiplexing step, amplicons are equimolarly pooled before being submitted to Illumina library construction and sequencing. Mastrevirus sequences identified using similarity search (sequences with green ticks on the figure) were more precisely classified through phylogenetic placement analysis on mastreviruses reference alignment and tree (in grey). Viral classifications are then confirmed after cloning and Sanger sequencing using the RCA-RFLP protocol.

A metagenomic approach based on RCA-Random PCR amplification tagging (RCA-RA-PCR) followed by a high throughput sequencing was developed (Fig. 1). Total genomic DNA was extracted from dried leaf material using the DNeasy Plant DNA extraction kit (Qiagen, USA) according to the manufacturer’s instructions and was then stored at −20 °C before use. RCA was carried out on each DNA extract using the Illustra Templiphi Kit (GE Healthcare, USA). A random amplification step using Polymerase Chain Reaction (RA-PCR) was then performed on diluted RCA products. This relied on the use of random primers each having at its 5′ extremity a single barcode of eight nucleotides, followed by six random nucleotides and the nucleotide motif TGGC (5′-BARCODE(8nt)-NNNNNN-TGGC-3′). A total of 160 barcodes previously defined^18^ with an edit distance of three were used, meaning that a minimum of three single-nucleotide changes (insertions, deletions or substitutions) are required for one barcode to be confound with another. Each sample was subjected to two independent random PCR reactions using two distinct random primers. In a total volume of 25 µl, 1 µl of diluted RCA DNA (1:10) was mixed with 5 µl of 5X buffer, 1 µl of dNTP (2 mM), 2.5 µl of MgCl2 (1.25 mM), 10 µl of random primers (4.5 µM) and 0.25 µl of GoTaq polymerase (Promega, USA). After an initial denaturation step at 94 °C for 3 minutes, 35 PCR cycles (at 94 °C for 1 minute, 50 °C for 1 minute and 72 °C for 1 minute) were carried out before a final elongation step for 5 minutes at 72 °C.

To enable equimolar pooling of the RA-PCR products, hereafter called amplicons, quantification was carried out using the Quant-iT PicoGreen dsDNA Assay Kit (Thermo Fisher Scientific, USA). Along with standards (eight duplicates of a DNA standard ranging from 0 to 40 ng/µl), 2 µl of diluted amplicons (1:4) were added to 98 µl of Picogreen mix (diluted to 1/200 with Tris-EDTA 1 × , supplied by the manufacturer) within a Fast Optical 96-well reaction plate (Applied Biosystems®). After vortexing and centrifugation at 600 rpm, plates were incubated in the dark at room temperature for five minutes. Fluorescence measurements were performed at 25 °C using a StepOnePlus Real-Time PCR system (Applied Biosystem, USA) at the excitation and emission wavelengths of Picogreen (respectively 480 nm and 530 nm). DNA concentrations of amplicons were obtained based on the fluorescence curve of the standard. It is important to notice here that the amount of DNA obtained from each PCR reaction is mostly independent of the infection status of the sample and that all amplicons were used for downstream experiments.

The 288 amplicons (two PCR replicates for 144 samples) obtained in the study were combined in pools (with up to 160 amplicons per pool) with a maximum concentration ratio of 1.5 (*i*.*e*. no amplicons could have a concentration more than 1.5 times higher than any one of the others in the same pool). Amplicon pools were then purified using the Illustra GFX^TM^ PCR DNA kit and gel purified (GE Healthcare, USA) according to the manufacturer’s instructions. Once purified, the pools were quantified using the Qubit dsDNA BR Assay Kit for the Qubit fluorometer (Thermo Fisher Scientific, USA) and checked on D5000 ScreenTape for 2200 TapeStation (Agilent Technologies, USA). Amplicon sizes ranged mostly from 300 bp to 5 kb. Amplicon pools were submitted to Illumina library construction and 2 × 250 bp paired-end sequencing on a Illumina HiSeq2500 sequencer at Genewiz (USA). A Covaris shearing step was performed to fragment amplicons to the desired size suitable for Illumina sequencing (between 200 and 700 bp) prior to library construction.

### Metagenomic data analyses

A quality control step (sliding window of 30 bases with an average quality of 25 bases required) was first performed on raw Illumina reads using Trimmomatic^19^ which was also used to remove Illumina adapter sequences (with a maximum mismatch of 2 bases, a palindromic match threshold in the range of 30 bases and a simple clip threshold of 10 bases). Sequences shorter than 80 nucleotides and unpaired reads were discarded. Trimmed reads were demultiplexed with exact matches using SABRE (https://github.com/najoshi/sabre). It is important to note here that, due to the Covaris shearing step, not all the amplicon pairs had an identifiable barcode on one or both reads. Pairs without any barcode were discarded from the analysis. After demultiplexing and primer sequence removal, overlapping paired-end reads were merged using PANDAseq^20^ and non-overlapping paired-end reads were abutted. Merged and abutted sequences were then dereplicated using VSEARCH v1.9.5^21^.

### Taxonomic assignment of sequences

An initial fast taxonomic assignment of dereplicated sequences was performed using Kraken v2^22^ with a custom database. This database was built with a maximum file size of 20 GB using the “max db size” option of “kraken2-build” from nucleotide sequences of archaeal, bacterial, plasmid, viral, fungal, plant, protozoan and human complete genomes within the NCBI Reference Sequence (RefSeq) and environmental sequencing project (env_nt) databases.

More precise classification of viral sequences, was achieved with similarity searches of both the viral RefSeq database using the algorithm in DIAMOND v0.9.19.120^23^, and on a database containing only geminivirus and geminivirus satellite sequences using the “usearch_global” algorithm of VSEARCH. Both databases were obtained from GenBank in October 2017. To reduce the dataset size, reads with similarities to geminivirus sequences were clustered using SWARM v2.1.9^24^ with the distance parameter set to 3.

Non-singleton clusters of mastrevirus sequences *i*.*e*. clusters gathering more than one read, were further classified with the phylogenetic placement approach implemented in pplacer (v1.1.alpha18-2-gcb55169)^25^. This phylogenetic placement method relies on a phylogenetic-based maximum-likelihood classification of sequences over a taxonomically informed reference alignment and tree which are combined into a so-called “reference package”. To construct such a package, all mastrevirus complete genome sequences along with their taxonomic information were obtained from GenBank in April 2018. After linearization of the circular sequences at the virion strand origin of replication and alignment using MAFFT v7.310^26^, the dataset was reduced using T-COFFEE v11^27^ to the ten most informative sequences for each mastrevirus species. Note that because of the circular nature of mastrevirus genomes, the reference sequences were tandemly repeated to avoid biases in the alignment of metagenomic sequences that traversed the virion strand origin of replication. A maximum-likelihood phylogenetic tree was then constructed using FastTree v2.1.8^28^. The reference package was built using taxtastic v0.6.4 (https://github.com/fhcrc/taxtastic) and evaluated using rppr v1.1^25^. After alignment of the merged sequences to the reference package alignment using the “addfragments” option of MAFFT, the phylogenetic placement was performed using pplacer with default parameters and sequence classifications obtained using rppr and guppy v1.1^25^. These classifications were analysed using the BoSSA R package (https://cran.r-project.org/package=BoSSA). Groups of samples with similar viral infection profiles were obtained after the clustering of the Kantorovich–Rubinstein distance matrix directly obtained from the placement files using guppy.

### Cloning and full genome sequencing

Representative samples from each viral infection profile group were selected for full genome cloning and sequencing. Full mastrevirus genomes were obtained using a RCA-RFLP based approach as previously described^29^. Briefly, 1 µl of RCA PCR was digested using *Bam*HI, *Eco*RI or *Nco*I to yield a ~2.7 kb fragment before purification using the Illustra GFX PCR DNA and Gel Band Purification Kit (GE Healthcare) according to the manufacturer’s instructions. The resulting purified fragments were ligated to the pJET 1.2 cloning vector (Thermo Fisher Scientific) and used to transform competent *Escherichia coli* (JM109, Promega). Recombinant plasmids were purified using QIAprep Spin Miniprep Kit (Qiagen) and were Sanger sequenced by Macrogen Inc. (Netherlands) using primer walking. Full-length mastrevirus genomes were then assembled with Geneious v6.0.6 (http://www.geneious.com)^30^.

### Phylogenetic and recombination analyses

Full genome nucleotide sequences were subjected to a BLAST search of the NCBI nt database for preliminary species assignment. One sequence of each mastrevirus species and all the genomes previously characterized from Reunion Island were selected from GenBank. These representative mastrevirus sequences, the sequences from Reunion Island and the full genome sequences determined in this study were linearized at the virion strand origin of replication and aligned using MAFFT. Pairwise similarities between the sequences were determined using SDT v1.2^31^. Recombination events were detected within the full genome sequences from Reunion Island using the RDP^32^, GENECONV^33^, BOOTSCAN^34^, MAXCHI^35^, CHIMERA^36^, SISCAN^37^ and 3SEQ^38^ methods included in the RDP4^39^ program. Default settings were used and recombination events were considered significant when detected by at least three methods. Maximum-likelihood phylogenetic trees were constructed using FastTree v2.1.8^28^ and were edited using the APE^40^ R package.

### Host identification

Sequencing of the *matK* and *rbcL* genes were performed on plant samples for which no confident genus identification could be achieved by visual inspection. PCRs were conducted before direct Sanger sequencing by Macrogen Inc. (Netherlands) as described previously^41^. After quality control, sequences were classified to the genus level using the RDP^42^ classifier against a database of *matK* and *rbcL* plant sequences obtained from GenBank.

## Results and Discussion

### Raw reads and the proportion of viral sequences

After quality control and barcode searches, 13 million sequence reads remained. The number of sequences obtained for the 144 Poaceae samples ranged from ~1000 to ~390000 with a mean of 108000. A first taxonomic assignment using Kraken revealed that 7% of the reads were detectably homologous with viral sequences, 3% with bacterial sequences and 28% with plant sequences. The remaining 62% of the reads were not detectably homologous to any sequences in the database. This high proportion of unclassified “dark matter” reads emphasises the large gap between the organisms which are known and those which remain to be discovered^43^. Among the reads likely to be of viral origin, 99% were most closely related to members of the genus *Mastrevirus*, 0.08% to geminivirus-associated satellites, 0.01% to members of the family *Genomoviridae* and the remaining 0.91% to members of other viral families. Importantly, no single sample could be associated with a high number of reads classified as geminivirus-associated satellites making these classifications mostly spurious.

### The Poaceae hosts of mastreviruses

To minimize the impact of contamination and sequencing errors when determining whether to flag a plant sample as being infected by a mastrevirus, it was necessary to set a threshold number of mastrevirus-derived reads above which a plant would be considered as being infected. If this threshold was set to 100 reads, the number of mastrevirus-infected plants in the sample was 23 (15%) whereas if the threshold was set to 1000 the number of infected plants decreased to 16 (11%). This article focusses on the samples determined to be infected using the more conservative threshold (*i*.*e*. 1000).

The sixteen mastrevirus-infected plants represent eight Poaceae species: *Cenchrus echinatus* (n = 1/1); *Cynodon dactylon* (n = 1/12); *Dactyloctenium aegyptium* (n = 1/4); *Digitaria ciliaris* (n = 6/27); *Eleusine indica* (n = 1/5); *Melinis repens* (n = 2/21); *Urochloa maxima* (n = 1/31) and *Sorghum arundinaceum* (n = 3/17) (Table 1). It is important to note here that whereas the two symptomatic *D*. *ciliaris* plants had clear evidence of the type of foliar streaking that is characteristic of AfSV infections in grasses, all 14 other infected plants did not present with any discernible streak symptoms. Therefore, in concordance with previous plant virus metagenomic studies, most non-cultivated plants that are infected by viruses appear to show no obvious outward signs of infection^10,11^: a factor emphasizing the importance during plant viral metagenomics studies of sampling plants regardless of their apparent health status.

**Table 1:** Summary of sampled plants and viral assignments.

Plant species	Number of samples	Number of positive samples	Viral profile	Mastrevirus species				
				MSV	MSRV	EIAV	SAAV	MeRAV
*Brachiaria umbellata*	1	0						
*Cenchrus echinatus*	1	1	2	1 (1)	1 (1)			
*Chloris gayana*	15	0						
*Cynodon dactylon*	12	1	1	1				
*Cyperus rotondus*	1	0						
*Dactyloctenium aegyptium*	4	1	1	1 (1)				
*Digitaria ciliaris*	27	6	1,2	6 (2)	1			
*Eleusine indica*	5	1	3			1 (1)		
*Melinis repens*	21	2	5					2 (1)
*Urochloa maxima*	31	1	4				1	
*Paspalum dilatatum*	1	0						
*Setaria pumila*	8	0						
*Sorghum arundinaceum*	17	3	4				3 (2)	
Total	144	16	5	9 (4)	2 (1)	1 (1)	4 (2)	2 (1)

The number in brackets refers to the number of full complete cloned genomes.

Of the eight mastrevirus-infected Poaceae species, five have already been described as mastrevirus hosts either on Reunion Island (*C*. *echinatus* and *D*. *ciliaris*) or elsewhere (*C*. *dactylon*, *E*. *indica* and *U*. *maxima*). The remaining three species, *D*. *aegyptium, M*. *repens and S*. *arundinaceum* have not previously been identified as mastrevirus hosts.

### Molecular characterization of mastreviruses

The most common approach that is presently used to taxonomically assign viral Illumina sequencing reads is the use of pairwise similarity searches of annotated sequences within public reference databases. Generally, these similarity searches employ BLAST or BLAST-like algorithms. When close relatives of the viral sequence reads are in the database, it is possible to assign the reads to a specific taxonomic rank. However, besides the difficulty of interpreting E-values and converting these to genetic distance measurements so that sequences can be assigned to an appropriate taxonomic rank, BLAST and BLAST-like algorithms can yield high miss-assignment rates when the reference database is inappropriate or incomplete (*i*.*e*. does not contain sequences that are closely related to the reads^44^).

Phylogenetic placement is an alternative to similarity search-based methods of read assignment, which attempts to map query sequences to a fixed reference phylogenetic tree according to a model of evolution using maximum likelihood (ML), Bayesian or neighbour joining (NJ) methods^45^. Ideally a query sequence will be placed on a branch of the phylogenetic tree at the exact location where it would branch on the tree had it been used along with the reference sequences during tree construction. Therefore, if a query sequence is very similar to one or more of the reference sequences used to construct the tree then it should be placed on a branch within a clade close to the tips of the tree that contains its closest relatives. On the other hand, a divergent query sequence which has no close relatives among the reference sequences, should be assigned to a branch that is close to the base of the tree.

**Figure 2:**
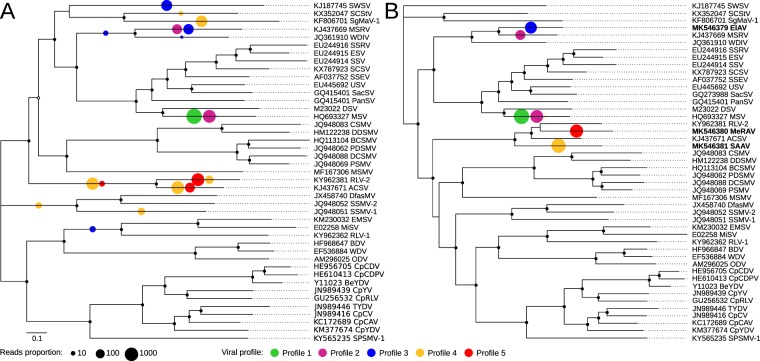
Phylogenetic placements of Illumina reads in a simplified Maximum-likelihood (ML) phylogenetic tree representing the breadth of known mastrevirus diversity. The ML phylogenetic tree was constructed from the complete genomes of 43 mastrevirus species (**A**) and three new mastreviruses cloned in this study (**B**). Open and closed circles on nodes indicate bootstrap support for the branches to their left of 70–89% and > = 90% respectively. Phylogenetic placements are summarised with coloured circles on branches whose sizes are proportional to the number of sequencing reads it represent and colours are function of the infection profile. More detailed phylogenetic placement trees are available in Supplementary Figures S1 and S2 and complete names of mastrevirus species are available in Supplementary Table S2.

After phylogenetic assignment of the mastrevirus-derived sequence reads, the sixteen positive plant samples were grouped into five infection profiles, defined as group of samples which present with similar viral taxonomic assignments. Two infection profiles correspond with samples having sequences clearly assigned to known mastrevirus species (placed close to the tips, profiles 1 and 2) and three with sequences assignments indicative of infection with unknown mastreviruses (placement scattered on the tree and including several basal branches, profiles 3, 4 and 5; Fig. 2A and Supplementary Figure S1). The first infection profile (green spots) correspond to seven samples infected exclusively with MSV strains: *C*. *dactylon* (n = 1), *D*. *aegyptium* (n = 1) and *D*. *ciliaris* (n = 5). The second profile (purple spots) corresponds to two samples co-infected by MSV and MSRV strains: *C*. *echinatus* (n = 1) and *D*. *ciliaris* (n = 1). The seven other samples yielded assignments suggestive of novel mastrevirus species in that no species-level virus assignments were evident following the phylogenetic placement of reads from these samples: *E*. *indica* (n = 1; profile 3, blue spots); *U*. *maxima* (n = 1) and *S*. *arundinaceum* (n = 3; profile 4, yellow spots) and *M*. *repens* (n = 2; profile 5, red spots). Importantly, virus detection and identification was effective and was congruent between replicates for 12 out of the 16 positive samples. In the four remaining samples the the virus was detected and identifiedin only a single replicate.

**Figure 3:**
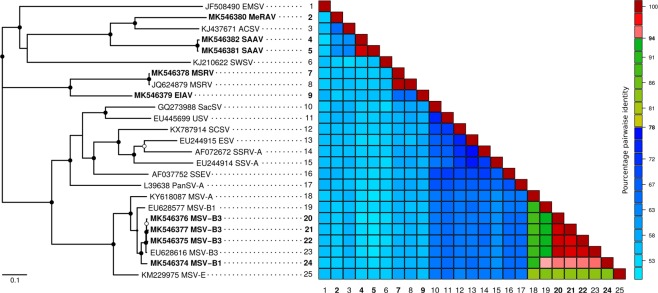
Maximum-likelihood phylogenetic tree (A) and pairwise sequence similarity matrix (B) of 16 known complete genomes of African streak viruses and the eight complete genomes determined in this study (indicated in bold font). The branches of the maximum likelihood tree are coloured according to geographical origins of the samples. Open and closed circles on nodes indicate bootstrap support for the branches to their left of 70–89% and > = 90% respectively. Complete names of mastrevirus species are available in Supplementary Table S2.

To confirm the MSV-B assignments indicated by profile 1, three complete genomes were cloned and sequenced from *D*. *aegyptium* (n = 1) and *D*. *ciliaris* (n = 2) plants with profile 1 (Table 1). A complete genome obtained from *D*. *aegyptium* (n = 1, [Reunion-Bassin Plat-Dactyloctenium aegyptium-RE025-2014], accession no. MK546377) and another from *D*. *ciliaris* (n = 1, [Reunion-Bassin Plat-Digitaria ciliaris-RE019-2014], accession no. MK546376) share between 98.7 and 99.4% identity with previously determined MSV-B3 sequences. A third genome from another profile 1 *D*. *ciliaris* plant ([Reunion-Bassin Plat-Digitaria ciliaris-RE081-2014], accession no. MK546374) shares 94.1% identity with previously determined MSV-B1 sequences (Fig. 3).

Similarly, the phylogenetic assignments that were made for the profile 2 samples – which indicate the presence of mixed MSV-B3 and MSRV infections – were validated by cloning one full MSV-B3 genome ([Reunion-Bassin Plat-Cenchrus echinatus-RE001-2014], accession no. MK546375) and one full MSRV genome ([Reunion-Bassin Plat-Cenchrus echinatus-RE001-2014],accession no. MK546378) from a profile 2 *C*. *echinatus* plant (Table 1). The cloned MSV-B3 genome shared 99.1% identity with previously determined MSV-B3 sequences and the cloned MSRV genome shared 99.8% identity with a previously determined MSRV-A sequence (Fig. 3).

MSV-B has previously been found infecting *C*. *echinatus* and *D*. *ciliaris*^12^, but this is the first time it has been characterized in *D*. *aegyptium* and *C*. *dactylon*. MSRV has previously been isolated in diverse Poaceae species (*Rottboellia* sp.*, Setaria barbata* and *Zea mays)* but has not previously been identified in *C*. *echinatus*. It must be noted here that the phylogenetic placement results for these mixed infections could not exclude the possibility that the apparent mixed infections in profile 2 plants could have been a consequence of these plants being infected by a recombinant of MSV and MSRV (rather than being attributable to a co-infection). However, our recovery of non-recombinant MSV-B and MSRV genomes from one of the profile 2 plants indicates that, even if MSV-MSRV recombinants occur in these plants, they are unlikely to represent a majority of the mastrevirus sequences within these plants.

Further, to confirm that the profiles 3, 4 and 5 samples did indeed contain novel mastreviruses, full length virus genomes were cloned and sequenced from at least one plant with profiles 3, 4 and 5 (Fig. 2B and Supplementary Figure S2): one from the *E*. *indica* (profile 3, blue spot, [Reunion-Bassin Plat-RE004-2014], accession no. MK546379), two from *S*. *arundinaceum* (profile 4, yellow spot, [Reunion-Bassin Plat-RE034-2014], [Reunion-Bassin Plat-RE084-2014], accession no. MK546381 and MK546382 respectively) and one from *M*. *repens* (profile 5, red spot, [Reunion-Bassin Plat-RE027-2014], accession no. MK546380; Table 1). When these newly determined sequences were included in the reference package and another round of phylogenetic placement performed (Fig. 2B and Supplementary Figure S2) all the reads of each of the seven profile 3, 4 and 5 samples were clearly assigned to clades containing the newly sequenced genomes. This indicated both that the cloned genomes do indeed represent all of the reads that were poorly assigned (placed on basal branches of the tree) during the first round of phylogenetic placement, and that there were no additional divergent mastrevirus sequences coinfecting the profiles 3, 4 and 5 plants.

The novel mastrevirus genome from *E*. *indica* ([Reunion-Bassin Plat-RE004-2014], accession no. MK546379) shares 65.6% nucleotide sequence identity with MSRV-A, that from *S*. *arundinaceum* ([Reunion-Bassin Plat-RE034-2014], [Reunion-Bassin Plat-RE084-2014], accession no. MK546381 and MK546382 respectively) shares 61.8–62.4% identity with *Axonopus compressus streak virus* (ACSV), and that from *M*. *repens* ([Reunion-Bassin Plat-RE027-2014], accession no. MK546380) shares 66.2% identity with ACSV (Fig. 3). Based on the current ICTV recommended pairwise similarity-based species demarcation threshold for mastreviruses (<78% identity^12^), these viruses therefore represent three novel mastrevirus species. The names *Eleusina indica associated virus* (EIAV)*, Sorghum arundinaceum associated virus* (SAAV) and *Melinis repens associated virus* (MeRAV) were proposed for these putative species.

A maximum-likelihood (ML) phylogenetic tree constructed with these three sequences and a representative selection of other mastreviruses (Fig. 3) confirms the pairwise sequence analysis results that EIAV is most closely related to MSRV and that both SAAV and MeRAV are most closely related to ACSV. It also confirms that these three novel mastreviruses fall within the AfSV group. EIAV, SAAV and MeRAV have a typical mastrevirus genome architecture. However, whereas the EIAV and SAAV sequences contain a canonical geminivirus TAATATTAC nonanucleotide sequence at the probable origin of virion-strand DNA replication, the MeRAV genome has an unusual TAACATTGC nonanucleotide motif.

**Figure 4:**
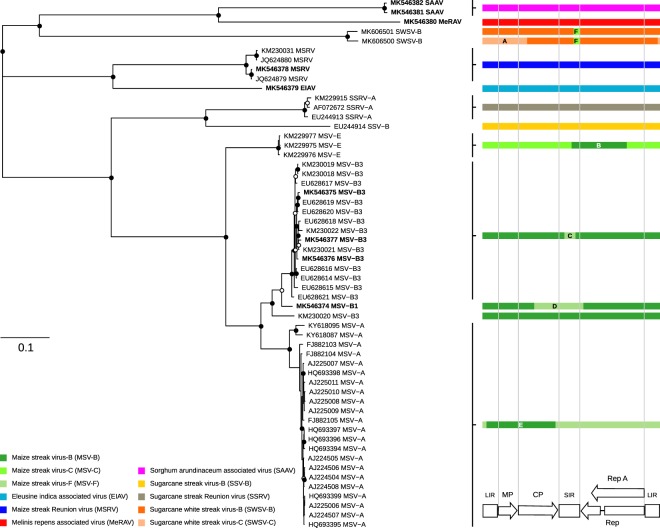
Phylogenetic relationships and recombination patterns among the AfSV species on Reunion Island. The maximum-likelihood phylogenetic tree contains 47 known complete genomes of monocot-infecting mastreviruses from Reunion Island and eight complete genomes determined in this study (indicated in bold font). The tree was rooted on chickpea chorosis virus (JN989413) as an outgroup (not shown). Open and closed circles on nodes indicate bootstrap support for the branches to their left of 70–89% and > = 90% respectively. The schematic representation of recombination events detected by RDP4 using seven different methods: RDP, GENCONV, BOOTSCAN, MAXCHI, CHIMERA, SISCAN and 3SEQ. Arrows and blocks at the bottom correspond respectively to open reading frames (ORFs) and intergenic regions: movement protein (*MP*), coat protein (CP), replication-associated proteins (Rep and Rep A), long intergenic region (LIR) and small intergenic region (SIR)The colours of blocks represent the different AfSV species and strains. More details on each event (lettered A to F) are available in Supplementary Table S3.

It is well known that intra- and inter-species recombination is very common in mastreviruses^42–44^. Because of the high degree of mastreviruses diversity on Reunion Island, all Reunion Island mastrevirus sequences were therefore analysed for evidence of recombination (Fig. 4, Supplementary Table S3). No recombination events were identified in the newly characterized MeRAV, SAAV and EIAV genomes. However, six recombination events were detected in other viruses: five of which were intra-species events (A to E in Fig. 4, Supplementary Table S3) in the *Sugarcane white streak virus* strain B (SWSV-B), MSV-E, MSV-B3, MSV-B1 and MSV-A sequences; and one of which was an inter-species event (F) in the SWSV-B sequence (Fig. 4, Supplementary Table S3). The inter-species recombination event was detected in the short intergenic region (SIR) whereas the intra-species recombination events were identified in the long intergenic region (LIR), SIR, the virion sense open reading frames (ORFs), MP and CP, and the C-terminus portion of Rep and Rep A ORFs. The LIR and SIR have previously been determined to be recombination hotspots in AfSVs^12,46^. Recombination events in SIR regions in MSV-B3 genomes (event C) and in MP and CP regions in MSV-A (event E) have been previously reported^12^. However, it’s the first description of recombination events in MSV-B1 (event D), SWSV-B (events A and F) and MSV-E (event B) in such genome regions (Fig. 4, Supplementary Table S3).

The inter-species recombination event involved a small genomic fragment transfer representing only 2.2% of the full genome size while the intra-species recombination events involved mainly large genomic fragment exchanges involving on average 24.7% of the full genome length. The sizes of these transferred genome fragments are on average larger than those previously detected in the genomes of mastreviruses infecting monocotyledonous plants^12,47,48^.

### Technical aspects with relevance for future studies of geminivirus diversity

From the perspective of future geminivirus diversity studies, it is noteworthy that the metagenomics approach used here permitted the pooling of 144 non-cultivated plants that were sampled at the scale of an individual farm. This degree of pooling, while higher than that used in previous plant metagenomics studies is still not at the upper limit of pooling that is possible using our approach. In fact, it should be possible to successfully pool up to 1,200 samples in a full Illumina sequencing lane. Given such dense pooling, our approach should be economical for preliminarily testing the infection status of large number of plants such as that required for plant quarantine testing and epidemiological surveillance. From the perspective of basic viral ecology research, our phylogenetics based taxonomic assignment approach should enable deep insights into the known and unknown geminiviral populations that are present within any given population of plants no matter the sampling scale.

It is important to stress, however, that there are also potential pitfalls associated with the use of both RCA and RA. Firstly, the RCA reaction can generate chimeric products that could lead to the detection of artifactual recombinants^49^. Second, RCA is not unbiased in its amplification of circular molecules and it should not be used to infer the actual relative frequencies of genetic variants in a population^50^. Third, RCA products were shown to be only representative of 0.05–0.06% of the initial DNA templates for another family of plant infecting circular ssDNA viruses and may therefore result in the detection of only the most common viruses within co-infections where there is a large numerical imbalance between the titres of the coinfecting virus genomes^51^. Fourth, the random primers used for RA possess a DNA clamp (TGGC) on the 3′ extremity that is intended to promote PCR efficiency. However, this clamp will reduce the randomness of the hybridization site and will therefore introduce biases with respect to both the genomic regions that are amplified most efficiently, and the viral genomic sequences that will be amplified most frequently. These biases, probably reduced with the low 50 °C annealing temperature during the PCR step, would impact the ability to detect viral sequences and the assembly of full genome sequences from short reads which could in turn reduce the accuracy with which sequences can be taxonomically assigned. Fifth, our approach is unable to constrain amplicon sizes and therefore requires a shearing step prior to Illumina library construction, which can result in many sequenced amplicons lacking one or both of their tags; many of these sequences must therefore be disregarded during downstream analyses.

An improvement of our approach would have been the inclusion of positive and negative controls within the sequencing reactions that could have been used to rationally determine a suitable read frequency threshold to defend against sequencing contamination. Because of the absence of such controls, an extremely conservative frequency threshold was applied and results from many samples that likely contained detectable mastrevirus sequences were disregarded. Optimising the read frequency threshold^52^ will be crucial for maximizing the sensitivity with which viruses can be detected within infected hosts without incurring an elevated false positive rate.

## Conclusions

The geminivirus-focused metagenomics approach presented here has proven useful in both the large-scale detection of know mastreviruses and the discovery of novel mastreviruses. It is ideally suited to studies focused on circular DNA viruses (such as geminiviruses and genomoviruses). It is unclear whether the large diversity of mastreviruses on Reunion Island is a consequence of the importation of these viruses to the island or whether some of these viruses might have originated on the island. Numerous crop, medicinal and ornamental plants have all been imported to these islands over the past centuries and it is entirely plausible that many different viruses could have been ferried to the island within these plants. Certainly, Reunion Island is a hotspot of mastrevirus diversity and it is possible that additional sampling on the island within a more diverse set of biomes will reveal even greater mastrevirus diversity than that encountered in this study. In this regard, newer “geo-referenced” metagenomic approaches (called geo-metagenomics), would be ideally suited to examining the relationships between land use history, plant distributions and viral diversity^53^.

## Supplementary information

Authors information
