# Supplementary material for: Exploring the diversity of Poaceae-infecting mastreviruses on Reunion Island using a viral metagenomics-based approach
Source: Sci Rep. 2019 Sep 3;9:12716. doi: 10.1038/s41598-019-49134-9 (PMC6722101; doi:10.1038/s41598-019-49134-9)
Supplement: Supplementary file 1 — Authors information [file 41598_2019_49134_MOESM1_ESM.docx]

Supplementary information

**Exploring the diversity of Poaceae-infecting mastreviruses on Reunion Island using a viral metagenomics-based approach**

Sohini Claverie, Alassane Ouattara, Murielle Hoarau, Denis Filloux, Arvind Varsani, Philippe Roumagnac, Darren P. Martin, Jean-Michel Lett, Pierre Lefeuvre^*^

^*^Corresponding author: Pierre Lefeuvre, [pierre.lefeuvre@cirad.fr](mailto:pierre.lefeuvre@cirad.fr)

CIRAD, UMR PVBMT, F-97410 St Pierre La Réunion France

+262262492719

**GenBank accession**

MSV-B1 [Reunion-Bassin Plat-Digitaria ciliaris-RE081-2014], MK546374

MSV-B3 [Reunion-Bassin Plat-Cenchrus echinatus-RE001-2014], MK546375

MSV-B3 [Reunion-Bassin Plat-Cenchrus echinatus-RE019-2014], MK546376

MSV-B3 [Reunion-Bassin Plat-Cenchrus echinatus-RE025-2014], MK546377

MSRV-A [Reunion-Bassin Plat-Cenchrus echinatus-RE001-2014], MK546378

EIAV [Reunion-Bassin Plat-RE027-2014], MK546379

MeRAV [Reunion-Bassin Plat-RE027-2014], MK546380

SAAV [Reunion-Bassin Plat-RE034-2014], MK546381

SAAV [Reunion-Bassin Plat-RE084-2014], MK546382

SWSV-B [Reunion-H11_R962569-2013], MK606500

SWSV-B [Reunion-HI46_R972341-2013], MK606501

**Supplementary Tables and Figures**

**Supplementary Table S1. Details of sampled plants from this study.**

| **Sample ID** | **Poaceae species** |
| --- | --- |
| RE001 | *Cenchrus echinatus* |
| RE002 | *Melinis repens* |
| RE003 | *Urochloa maxima* |
| RE004 | *Eleusine indica* |
| RE005 | *Digitaria ciliaris* |
| RE006 | *Melinis repens* |
| RE007 | *Setaria pumilata* |
| RE008 | *Sorghum arundinaceum* |
| RE009 | *Digitaria ciliaris* |
| RE010 | *Setaria pumilata* |
| RE011 | *Chloris* sp. |
| RE012 | *Urochloa maxima* |
| RE013 | *Paspalum dilatatum* |
| RE014 | *Eleusine indica* |
| RE015 | *Urochloa maxima* |
| RE016 | *Cynodon dactylon* |
| RE017 | *Digitaria ciliaris* |
| RE018 | *Digitaria ciliaris* |
| RE019 | *Dactyloctenium aegyptium* |
| RE020 | *Chloris* sp. |
| RE021 | *Urochloa maxima* |
| RE022 | *Cynodon dactylon* |
| RE023 | *Dactyloctenium aegyptium* |
| RE024 | *Eleusine indica* |
| RE025 | *Melinis repens* |
| RE026 | *Urochloa maxima* |
| RE027 | *Sorghum arundinaceum* |
| RE028 | *Digitaria ciliaris* |
| RE029 | *Setaria pumilata* |
| RE030 | *Digitaria ciliaris* |
| RE031 | *Setaria pumilata* |
| RE032 | *Melinis repens* |
| RE033 | *Urochloa maxima* |
| RE034 | *Sorghum arundinaceum* |
| RE035 | *Digitaria ciliaris* |
| RE036 | *Setaria pumilata* |
| RE037 | *Chloris* sp. |
| RE038 | *Chloris* sp. |
| RE039 | *Cynodon dactylon* |
| RE040 | *Zea mays* |
| RE041 | *Digitaria ciliaris* |
| RE042 | *Chloris africana* |
| RE043 | *Melinis repens* |
| RE044 | *Urochloa maxima* |
| RE045 | *Sorghum arundinaceum* |
| RE046 | *Digitaria ciliaris* |
| RE047 | *Melinis repens* |
| RE048 | *Urochloa maxima* |
| RE049 | *Digitaria ciliaris* |
| RE050 | *Chloris* sp. |
| RE051 | *Cynodon dactylon* |
| RE052 | *Melinis repens* |
| RE053 | *Urochloa maxima* |
| RE054 | *Digitaria ciliaris* |
| RE055 | *Chloris* sp. |
| RE056 | *Sorghum arundinaceum* |
| RE057 | *Digitaria ciliaris* |
| RE058 | *Dactyloctenium aegyptium* |
| RE059 | *Setaria pumilata* |
| RE060 | *Eleusine indica* |
| RE061 | *Melinis repens* |
| RE062 | *Urochloa maxima* |
| RE063 | *Sorghum arundinaceum* |
| RE064 | *Digitaria ciliaris* |
| RE065 | *Urochloa maxima* |
| RE066 | *Sorghum arundinaceum* |
| RE067 | *Digitaria ciliaris* |
| RE068 | *Digitaria ciliaris* |
| RE069 | *Melinis repens* |
| RE070 | *Urochloa maxima* |
| RE071 | *Cynodon dactylon* |
| RE072 | *Digitaria ciliaris* |
| RE073 | *Digitaria ciliaris* |
| RE074 | *Melinis repens* |
| RE075 | *Urochloa maxima* |
| RE076 | *Cynodon dactylon* |
| RE077 | *Setaria pumilata* |
| RE078 | *Urochloa maxima* |
| RE079 | *Sorghum arundinaceum* |
| RE080 | *Cynodon dactylon* |
| RE081 | *Digitaria ciliaris* |
| RE082 | *Melinis repens* |
| RE083 | *Urochloa maxima* |
| RE084 | *Sorghum arundinaceum* |
| RE085 | *Cynodon dactylon* |
| RE086 | *Melinis repens* |
| RE087 | *Urochloa maxima* |
| RE088 | *Sorghum arundinaceum* |
| RE089 | *Digitaria ciliaris* |
| RE090 | *Chloris* sp. |
| RE091 | *Melinis repens* |
| RE092 | *Urochloa maxima* |
| RE093 | *Sorghum arundinaceum* |
| RE094 | *Digitaria ciliaris* |
| RE095 | *Melinis repens* |
| RE096 | *Urochloa maxima* |
| RE097 | *Sorghum arundinaceum* |
| RE098 | *Digitaria ciliaris* |
| RE099 | *Melinis repens* |
| RE100 | *Urochloa maxima* |
| RE101 | *Sorghum arundinaceum* |
| RE102 | *Cyperus rotondus* |
| RE103 | *Eleusine indica* |
| RE104 | *Cynodon dactylon* |
| RE105 | *Digitaria ciliaris* |
| RE106 | *Chloris* sp. |
| RE107 | *Melinis repens* |
| RE108 | *Urochloa maxima* |
| RE109 | *Sorghum* sp. |
| RE110 | *Digitaria ciliaris* |
| RE111 | *Chloris* sp. |
| RE112 | *Melinis repens* |
| RE113 | *Urochloa maxima* |
| RE114 | *Chloris* sp. |
| RE115 | *Melinis repens* |
| RE116 | *Urochloa maxima* |
| RE117 | *Dactyloctenium aegyptium* |
| RE118 | *Chloris* sp. |
| RE119 | *Melinis repens* |
| RE120 | *Urochloa maxima* |
| RE121 | *Sorghum arundinaceum* |
| RE122 | *Digitaria ciliaris* |
| RE123 | *Chloris* sp. |
| RE124 | *Urochloa maxima* |
| RE125 | *Sorghum arundinaceum* |
| RE126 | *Digitaria ciliaris* |
| RE127 | *Brachiaria umbellata* |
| RE128 | *Urochloa maxima* |
| RE129 | *Cynodon dactylon* |
| RE131 | *Chloris* sp. |
| RE132 | *Cynodon dactylon* |
| RE133 | *Urochloa maxima* |
| RE134 | *Urochloa maxima* |
| RE135 | *Melinis repens* |
| RE136 | *Urochloa maxima* |
| RE137 | *Sorghum arundinaceum* |
| RE138 | *Melinis repens* |
| RE139 | *Urochloa maxima* |
| RE140 | *Digitaria ciliaris* |
| RE141 | *Chloris* sp. |
| RE142 | *Urochloa maxima* |
| RE143 | *Digitaria ciliaris* |
| RE144 | *Urochloa maxima* |
| RE145 | *Cynodon dactylon* |

**Supplementary Table S2. Summary of mastrevirus species and their acronyms.**

| **Virus specie** | **Acronym** |
| --- | --- |
| *Axonopus compressus streak virus* | ACSV |
| *Barley dwarf virus* | BDV |
| *Bean yellow dwarf virus* | BYDV |
| *Bromus catharticus striate mosaic virus* | BCSMV |
| *Chickpea chlorosis Australia virus* | CpCAV |
| *Chickpea chlorosis virus* | CpCV |
| *Chickpea chlorotic dwarf Pakistan virus* | CpCDPV |
| *Chickpea chlorotic dwarf virus* | CpCDV |
| *Chickpea redleaf virus* | CpRLV |
| *Chickpea yellow dwarf virus* | CpYDV |
| *Chickpea yellow virus* | CpYV |
| *Chloris striate mosaic virus* | CpSMV |
| *Digitaria ciliaris striate mosaic virus* | DCSMV |
| *Digitaria didactyla striate mosaic virus* | DDSMV |
| *Digitaria streak virus* | DSV |
| *Dragonfly-associated mastrevirus* | DfasMV |
| *Eleusina indica associated virus* | EIAV |
| *Eragrostis minor streak virus* | EMSV |
| *Eragrostis streak virus* | ESV |
| *Maize streak Reunion virus* | MSRV |
| *Maize streak virus* | MSV |
| *Maize striate mosaic virus* | MSMV |
| *Melinis repens associated virus* | MeRAV |
| *Miscanthus streak virus* | MiSV |
| *Oat dwarf virus* | ODV |
| *Panicum streak virus* | PanSV |
| *Paspalum dilatatum striate mosaic virus* | PDSMV |
| *Paspalum striate mosaic virus* | PSMV |
| *Rice latent virus 1* | RLV-1 |
| *Rice latent virus 2* | RLV-2 |
| *Saccharum streak virus* | SacSV |
| *Sorghum arundinaceum associated virus* | SAAV |
| *Sporobolus striate mosaic virus 1* | SSMV-1 |
| *Sporobolus striate mosaic virus 2* | SSMV-2 |
| *Sugarcane chlorotic streak virus* | SCSV |
| *Sugarcane streak Egypt virus* | SSEV |
| *Sugarcane streak Reunion virus* | SSRV |
| *Sugarcane streak virus* | SSV |
| *Sugarcane striate virus* | SCStV |
| *Sugarcane white streak virus* | SWSV |
| *Sweetpotato symptomless mastrevirus 1* | SPSMV-1 |
| *Switchgrass mosaic-associated virus 1* | SgMaV-1 |
| *Tobacco yellow dwarf virus* | TYDV |
| *Urochloa streak virus* | USV |
| *Wheat dwarf India virus* | WDIV |
| *Wheat dwarf virus* | WDV |

**Supplementary Table S3.** Details of the recombination events detected using RDP4. Each event is lettered (A to F) according to Figure 4. The potential major and minor parents indicate the approximate identities of parental sequences that respectively donated the larger and the smaller fraction of the recombinant’s genome. Breakpoint positions are relative to the recombinant sequence. Methods used to detect recombination events are as follow RDP (R), GENECONV (G), BOOTSCAN (B), MAXCHI (M), CHIMERA (C), SISCAN (S) and 3SEQ (T). The method with the most significant *p*-value is indicated in bold and the associated *p*-value is shown. An undetermined breakpoint position is represented by a star.

| **Event** | **Major parent(s)** | **Minor parent(s)** | **Breakpoints** | | **Methods** | ***p*-value** |
| --- | --- | --- | --- | --- | --- | --- |
|  |  |  | **Begin** | **End** |  |  |
| A | SWSV-B  KJ210622, KJ187747,  KJ187748, MK606501 | SWSV  KJ187749 | 21 | 601 | RGMCS**T** | 6.57 x 10^-28^ |
| B | MSV-C  KM230004 | MSV-B3  EU628621, EU628618,  KM230021, KM230022 | 1402* | 2058 | RMC**S**T | 2.57 x 10^-6^ |
| C | MSV-B1  MK546374 | MSV-F  KM229946,EU628628, EU628629,EU628630,  KJ437654, KJ437655,  KJ437656, KM229944,  KM229945, KM229947,  KM229948, KM229949,  KM229950, KM229951,  KM229952, KM229953,  KM229954, KM229955,  KM229956, KM229957,  KM229958, KM229959,  KM229960, KM229961,  KM229962, KM229963,  KM229964, KM229965,  KM229966, KM229967,  KM229968, KM229969,  KM229970, KM229971,  KM229972, KM229973,  KM229974 | 1204 | 1394 | **R**GBMCST | 2.84 x 10^-29^ |
| D | MSV-B3  MK546375, EU628620,  EU628619, EU628618,  KM230021, EU628614,  EU628616, KM230028,  EU628617, KM230018,  KM230019, KM230029,  KM230026, KM230027,  EU628615, EU628621,  KM230022, KM230024,  KM230025 | MSV-F  EU628628, EU628629,  KJ437656, KJ437654,  KJ437655, KM229973,  KM229974, KM229971  KM229972, KM229944,  KM229945, KM229949,  KM229948, KM229947,  KM229951, KM229952,  KM229956, KM229961,  KM229953, KM229963,  KM229954, KM229955,  KM229964, KM229946,  KM229950, KM229966,  KM229969, KM229968,  KM229958, KM229960,  KM229959, KM229962,  KM229965, KM229967,  EU628630, KM229970,  KM229957 | 1244 | 1708 | **R**GBMCS | 2.08 x 10^-16^ |
| E | MSV-F  KM229944, EU628627,  EU628628, EU628629,  KJ437656, KJ437654,  KJ437655, KM229973,  KM229974, KM229971,  KM229972, KM229945,  KM229949, KM229948,  KM229947, KM229951,  KM229952, KM229956,  KM229961, KM229953,  KM229963, KM229954,  KM229955, KM229964,  KM229946, KM229950,  KM229966, KM229969,  KM229968, KM229958,  KM229960, KM229959,  KM229962, KM229965,  KM229967, EU628630,  KM229970 | MSV-B  EU628621, KM230018,  KM230029, KM230026,  EU628615, KM230022,  MK546377, KM230023,  EU628602, EU628582,  EU628585, AF239962,  EU628580, EU628579,  EU628598, EU628578,  EU628604, EU628600,  EU628596, KY554667,  EU628581, KM230017,  AF329886, EU628595,  EU628597, KY554664,  EU628599, KY554660,  KY554668, KY554663,  KY554655, KY554656,  KY554657, KY554658,  KY554666, KY554662,  KY554661, KY554659,  KM230014, KM230016,  KM230015, EU152260,  EU628609, EU628577,  EU628588, EU628587,  EU152261, EU628589,  EU628603, EU628586,  EU628607, EU628645,  EU628590, EU628601,  EU628606, EU628605,  EU628592, EU628608,  AF329887, EU628610,  EU628611, KM230030,  EU628612, EU628613,  KM230020 | 33 | 1374* | RGBM**S**T | 2.41 x 10^-22^ |
| F | SWSV-B  KJ210622, KJ187747,  KJ187748, MK606501, | MSV-C  AF007881, KM229978,  KM229983, KM229984,  KM229985, KM229986,  KM229987, KM229988,  KM229989, KM229990,  KM229991, KM229992,  KM229993, KM229994,  KM229995, KM229996,  KM229997, KM229998,  KM229999, KM230000,  KM230001, KM230002,  KM230003, KM230004,  KM230005, KM230006,  KM230007, KM230008,  KM230009, KM230010,  KM230011, KM230012 | 1383 | 1449 | **R**GB | 3.60 x 10^-6^ |


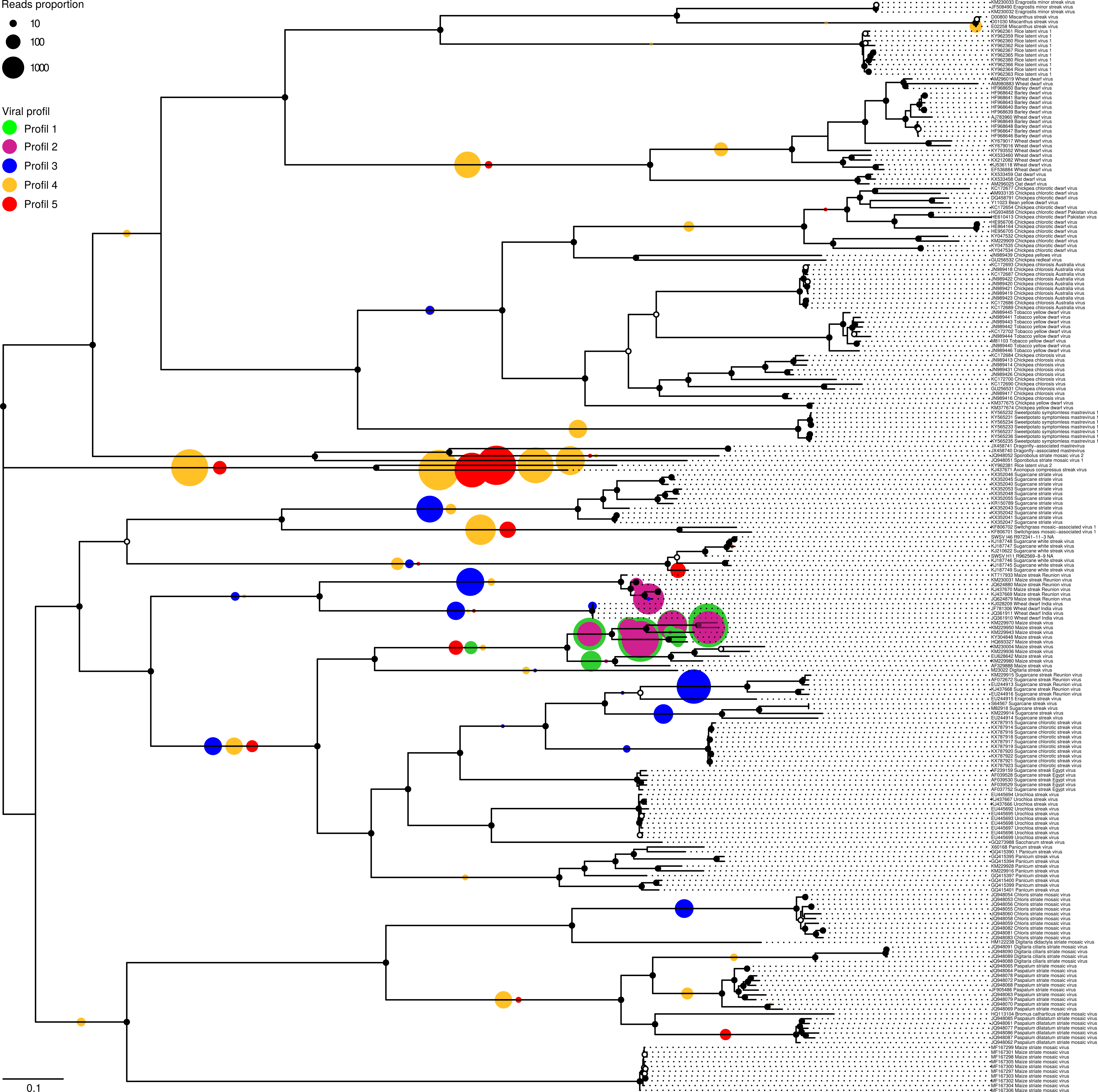


**Supplementary Figure S1.** Phylogenetic placements of Illumina reads in Maximum-likelihood (ML) phylogenetic tree representing known mastrevirus diversity, not including the new mastrevirus species characterized in this study. The ML phylogenetic tree was constructed with the ten most phylogenetically informative sequences for each mastrevirus species. Open and closed circles on nodes indicate bootstrap support for the branches to their left of 70-89% and >=90% respectively. Phylogenetic placements are summarised with colored circles on branches whose sizes are propotionnal to the number of sequencing reads it represent and colors are function of the infection profile.


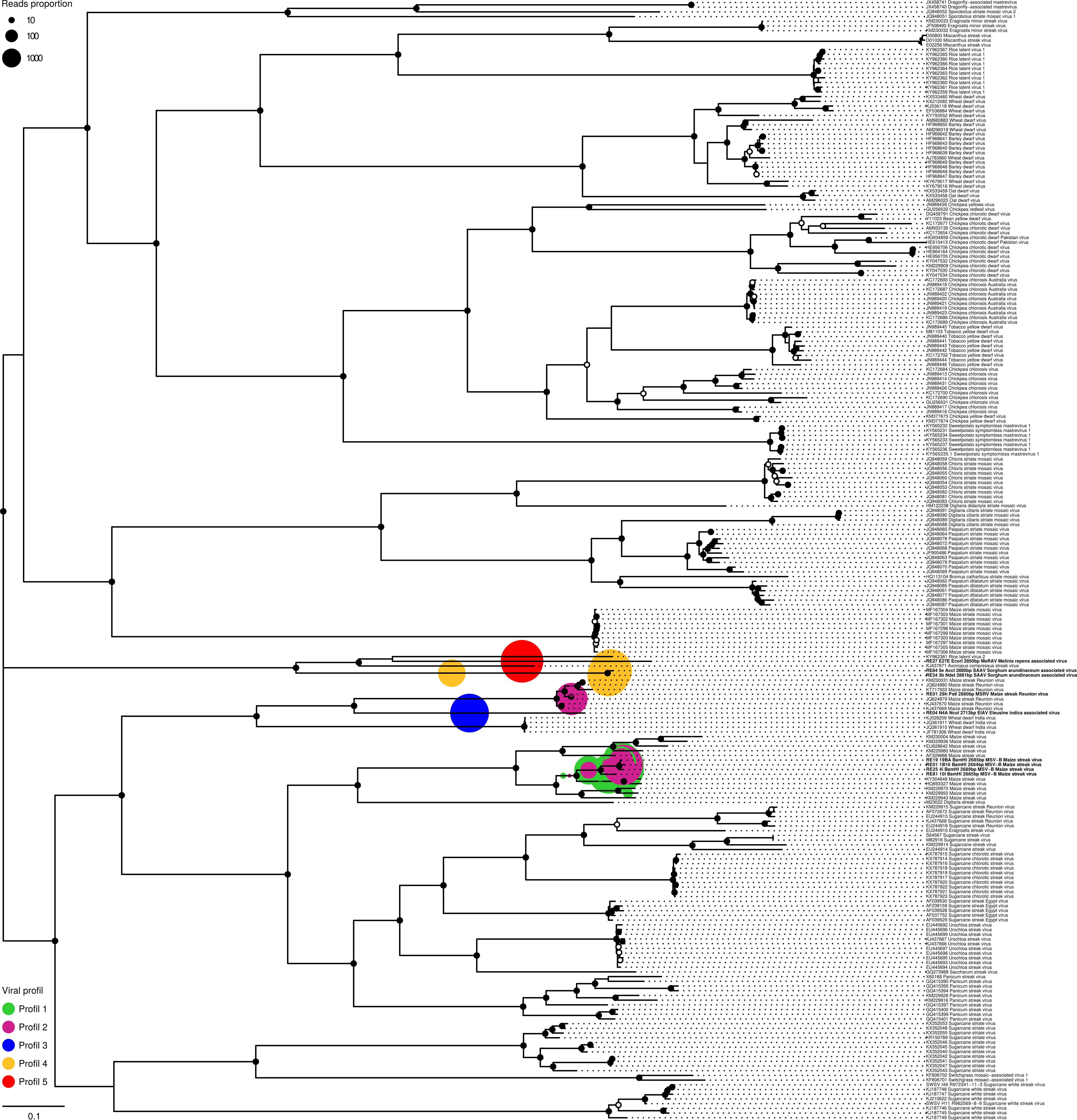


**Supplementary Figure S2.** Phylogenetic placements of Illumina reads in Maximum-likelihood (ML) phylogenetic tree representing known mastrevirus diversity, including the new mastrevirus species characterized in this study. The ML phylogenetic tree was constructed with the ten most phylogenetically informative sequences for each mastrevirus species and the three mastreviruses genome sequences determined in this study. Open and closed circles on nodes indicate bootstrap support for the branches to their left of 70-89% and >=90% respectively. Phylogenetic placements are summarised with colored circles on branches whose sizes are propotionnal to the number of sequencing reads it represent and colors are function of the infection profile.
